# Inflammation Factors and Genistein Supplementation in Cancer—Preliminary Research

**DOI:** 10.3390/cimb46030140

**Published:** 2024-03-07

**Authors:** Karolina Banyś, Małgorzata Jelińska, Małgorzata Wrzosek, Dorota Skrajnowska, Robert Wrzesień, Wojciech Bielecki, Barbara Bobrowska-Korczak

**Affiliations:** 1 Department of Toxicology and Food Science, Faculty of Pharmacy, Medical University of Warsaw, Poland, Banacha 1, 02-097 Warsaw, Poland; 2 Department of Biochemistry and Pharmacogenomics, Faculty of Pharmacy, Medical University of Warsaw, Poland, Banacha 1, 02-097 Warsaw, Poland; 3 Central Laboratory of Experimental Animals, Medical University of Warsaw, Poland, Banacha 1, 02-097 Warsaw, Poland; 4 Department of Pathology and Veterinary Diagnostics, Institute of Veterinary Medicine, Warsaw University of Live Sciences, Nowoursynowska 159c Street, 02-787 Warsaw, Poland

**Keywords:** genistein, cancer, nanoparticles

## Abstract

The purpose of this study was to evaluate the effect of genistein in nano, micro, and macro forms on the intensity of the DMBA-induced tumor process in rats and to understand the mechanisms of this action. The effect of genistein supplementation on the content of selected eicosanoids (HETEs, HODE, and HEPE) in the serum of rats was evaluated. The levels and expression of genes encoding various pro-inflammatory cytokines (IL-1, IL-6) and MMP-9 in the blood of rats were also investigated. The biological material for the study was blood obtained from female rats of the Sprague Dawley strain (n = 32). The animals were randomly divided into four groups: animals without supplementation, and animals supplemented at a dose of 0.2 mg/kg b.w. (0.1 mg/mL) with macro, micro (587 ± 83 nm), or nano (92 ± 41 nm) genistein. To induce mammary neoplasia (adenocarcinoma), rats were given 7,12-dimethyl-1,2-benz[a]anthracene (DMBA). The content of selected eicosanoids was determined by liquid chromatography with UV detection. An immunoenzymatic method was used to determine the content of cytokines and MMP-9. The expression of the *IL-6, IL-1beta,* and *MMP-9* genes was determined with quantitative real-time PCR (qRT-PCR) using TaqMan probes. Based on the study, it was shown that supplementation of animals with genistein in macro, micro, and nano forms increased the intensity of the tumor process in rats. It was shown that the content of 12-HEPE, HODE, and 12-HETE in the serum of genistein-supplemented rats was statistically significantly lower with respect to the content of the aforementioned markers in the serum of rats receiving only a standard diet, devoid of supplementation. It was found that animals supplemented with nano-, micro-, and macrogenistein had higher levels of metalloproteinase-9, MMP-9, compared to animals without supplementation. There was a significant increase in MMP-9 gene expression in the blood of macrogenistein-supplemented animals, relative to the other groups of rats. On the basis of the study, it was shown that supplementation of animals with nano-, micro-, and macrogenistein had an effect on the development of the tumor process. Dietary supplementation with genistein significantly decreased the level of selected eicosanoids, which may have significant impacts on cancer development and progression.

## 1. Introduction

In the search for compounds with anticancer effects, research into the use of genistein is receiving considerable attention. Genistein is included in the group of isoflavones. The main dietary source of genistein is soybeans and soy products. It is also found in alfalfa and lima sprouts, broccoli, cauliflower, and barley, among others [1,2]. It is worth noting that a number of preparations containing soy extracts and/or genistein itself are available in pharmacies. Numerous studies have shown that genistein can exhibit anticancer effects by, among other things, inducing apoptosis, affecting the cell cycle, inhibiting angiogenesis, and antiproliferative activity [3]. On the other hand, there are data supporting the pro-cancerous effects of genistein, especially in hormone-dependent cancer types [4,5,6,7,8]. One of the best-known properties of genistein is its estrogenic activity. Phytoestrogens have a chemical structure similar to 17β-estradiol and show the ability to bind to the estrogen receptor. Therefore, they can stimulate the process of carcinogenesis and increase the risk of breast cancer. Estrogens can exert carcinogenic effects through estrogen-receptor-dependent mechanisms, but also through the action of genotoxic products of estrogen metabolism [9]. The effect of genistein in the context of cancer therapy probably depends on the use of a specific dose, the physiological state of the test organism, and diet. We still do not know to whom, and in what doses, genistein should be administered to achieve the desired health effect, the mechanism of its action at the stage of initiation and progression of the cancer process is still unknown. It is also important to answer the question of whether it is safe for women to use supplements containing genistein in the context of cancer risk. Another important aspect is the answer to the question of how genistein in micro and nano forms might work. The studies on nanosized forms of phenolic compounds have become particularly important these days. The nano form changes their bioavailability. Bioavailability is defined in three basic steps: absorption, penetration into systemic circulation, and use in the cells. Reduction of materials at the nanoscale can lead to the development of new physical, chemical, and biological activities compared to the macro compounds [10]. It should be noted that there is still a lack of research in the literature in the field presented [10].

Therefore, the purpose of this study was to evaluate the effect of genistein in nano, micro, and macro forms on the intensity of DMBA-induced tumorigenesis. The effect of genistein supplementation on the content of selected eicosanoids (5-, 12-, and 15-hydroxyeicosatetraenoic acids (HETEs), 12-hydroxyeicosapentaenoic acid (HEPE), and the sum of hydroxyoctadecadienoic acids (HODE)) and levels and expression of genes encoding various pro-inflammatory cytokines (interleukin-1 (IL-1), interleukin-6 (IL-6)) and metalloproteinase-9 (MMP-9) in the blood of rats were also investigated. Despite numerous studies, the influence of genistein on these issues in the early stages of carcinogenesis is still not entirely clear.

## 2. Materials and Methods

### 2.1. Laboratory Animals

The biological material for the study was blood obtained from female rats of the Sprague Dawley strain (n = 32). Approval for the study was obtained from the Bioethics Committee at the Warsaw Medical University—document number 645/2018. During the experiment, the animals were housed under controlled conditions and provided with constant access to water and feed. They were fed Labofeed H standard diet (Labofeed H, Żurawia 19, 89-240 Kcynia, Poland). The room temperature was kept at 22 °C, and a 12-h day–night cycle was maintained.

The experiment lasted 100 days. After a 10-day period of adaptation to the experimental conditions, the animals were randomly divided into 4 groups:

Group 1—control group, rats without supplementation, which, in order to maintain the experimental conditions, received 0.4 mL of water via an intragastric probe.

Group 2—animals supplemented with macrogenistein, suspended in 0.4 mL of water, administered via an intragastric probe, at a dose of 0.2 mg/kg b.w. 

Group 3—animals supplemented with microgenistein, in the form of particles of 587 ± 83 nm, suspended in 0.4 mL of water, administered via an intragastric probe, at a dose of 0.2 mg/kg b.w. 

Group 4—animals supplemented with nanogenistein, in the form of 92 ± 41 nm particles suspended in 0.4 mL of water, administered via an intragastric probe at a dose of 0.2 mg/kg b.w. 

The procedure for preparing and evaluating the sizes of the nano- and micro-particles of genistein was presented in the work of Banyś et al. [11]. The dose of genistein used was based on the value of its average daily dietary intake by humans with extrapolation to the body weight of rats [12]. Rats were supplemented from 40 days of age until 20 weeks of age. 

### 2.2. Tumor Formation

To induce mammary tumorigenesis (adenocarcinoma), rats were twice administered 7,12-dimethyl-1,2-benz[a]anthracene (DMBA) (from Sigma-Aldrich, St. Louis, MO, USA) dissolved in rapeseed oil by intragastric probe. The first dose—80 mg/kg b.w.—was administered at day 60 of each animal’s life, and the next dose—40 mg/kg b.w.—at day 90 of each animal’s life. Examining the development and expansion of tumor nodules was carried out through manual examination. The rats were sacrificed at 150 days old, and an assessment was made regarding the quantity of tumors per rat as well as the weight of each tumor.

### 2.3. Histopathology

The tumors were placed in a buffered formalin solution, dehydrated, sealed in paraffin and cut into 4 μm thick sections. Hematoxylin and eosin staining of tissue and cell sections was applied. Evaluation of the stained sections was conducted using a BX43 Olympus research microscope (Olympus Europa SE & Co., Hamburg, Germany). Mitoses were quantified in slides sourced from randomly chosen tumors, examining 15 fields of view under a 40× objective magnification.

### 2.4. Determination of 5-, 12-, and 15-Hydroxyeicosatetraenoic Acids (5-, 12-, and 15-HETE), 12-Hydroxyeicosapentaenoic Acid (12-HEPE), and Hydroxyoctadecadienoic Acids (HODE) in Rat Serum

Serum was obtained from fresh blood (collected when the animals were killed) by centrifugation in a centrifuge at 3000 rpm at 4 °C. The biological material was stored in a low-temperature refrigerator at −70 °C until proper analyses were performed.

The contents of 5-, 12-, and 15-hydroxyeicosatetraenoic acids (5-, 12-, and 15-HETE), 12-hydroxyeicosapentaenoic acid, and hydroxyoctadecadienoic acids (HODE) in the serum of rats supplemented with nano-, micro-, and macrogenistein were determined using high-performance liquid chromatography with UV detection (HPLC/UV) based on the methodology developed by Frohberg et al. [13].

Bakerbond C18 500 mg/3 mL columns (from SPE, J.T. Baker, The Netherlands) were used to extract the 5,12,15-HETE, 12-HEPE, and HODE acids. The column bed was preconditioned with methanol, followed by water at 10 mL per column. A volume of 0.5 mL of 10% methanol was added to the serum sample (0.4 mL) and then applied to the column. After washing with 2 mL of water and 2 mL of 10% methanol, the test compounds were eluted with pure methanol (100%) (3 × 0.5 mL). The samples were evaporated to dryness under a stream of nitrogen at 37 °C and dissolved in 100 μL of ethanol. Before being applied to the chromatography column, each sample was cleaned using a 0.22 μm pore-size filter (Ultrafree-MC, Durapore PVDF, 0.22 μm, from Millipore, Burlington, MA, USA). The content of fatty acid metabolites was determined using a liquid chromatograph with an LC-20AD-type pump, SPD-10AV UV/VIS, detector and CT0-10AS oven (from Shimadzu Corporation, Kyoto, Japan). The tested compounds were separated on a 2.6 μm, 100 mm × 4.6 mm C18 Kinetex-type chromatography column (from Phenomenex, Torrance, CA, USA). The analysis was carried out using the following phase systems: A—methanol: acetic acid (100:0.01); and B—0.01% acetic acid. The composition of the mobile phase (A:B) changed over time as follows: 70:30 at 11 min; 73:27 at 18 min; 90:10 at 25 min, 70:30 at 5 min. The flow rate was 0.8 mL/min, and the column temperature was 35 °C. A 4 µL sample was applied to the chromatography column. The content of fatty acid metabolites was determined at 235 nm. Analysis of the tested compounds was carried out using standard solutions of HODE, 12-HEPE, 5-, 12-, 15-HETE (from Cayman Chemicals, Ann Arbor, MI, USA).

### 2.5. Determination of the Contents of Interleukin-1, Interleukin-6, and Metalloproteinase-9 in the Serum of Rats

The contents of interleukin-1, interleukin-6, and metalloproteinase-9 in the serum of rats supplemented with nano-, micro-, and macrogenistein were determined using ELISA. Commercially available assays were used for the study: Rat Interleukin-6 (IL-6) ELISA Kit (catalog number: orb219833) and Rat Interleukin-1 (IL-1) ELISA kit (catalog number: 219820) from Biorbyt (Biorbyt^®^ Ltd., 5, Orwell Furlong, Cowley Road, Cambridge, Cambridgeshire, CB4 0WY, UK); and Rat Matrix Metalloproteinase-9 ELISA Kit (catalog number: E0553r) from Wuhan Eiaab Science Co., Ltd. (Wuhan Eiaab Science Co., Ltd., Beneficiary, A1710 Guangguguoji, East Lake Hi-Tech Development Zone, Wuhan 430079, China). In performing the test, the test procedure was followed in accordance with the manufacturer’s instructions. A spectrophotometer from Bio-Tek Instruments (JNC, Highland Park, Box 998, Winooski, VT 05404-0998, USA) was used for the determination of IL-6, IL-1, and MMP-9.

### 2.6. Determination of Gene Expression Activity of Interleukin-1, Interleukin-6, and Metalloproteinase-9 in Rat Blood

The expression of *IL-6, IL-1beta* and *MMP-9* genes was determined with quantitative real-time PCR (qRT-PCR) using TaqMan probes. RNA was extracted using a Total RNA Mini kit (A&A Biotechnology, Gdynia, Poland) according to the manufacturer’s instruction. The RNA concentration and the purity were evaluated with a micro-volume UV–vis spectrophotometer (Quawell Q3000, Quawell Technology Inc., San Jose, CA, USA). RNA was reverse transcribed to cDNA with a High-Capacity RNA-to-cDNA Kit (Applied Biosystems, Waltham, MA, USA). TaqMan™ Gene Expression Master Mix was used in the research (catalogue no.: 4369016; Thermo Fisher Scientific, Inc., Waltham, MA, USA).

The TaqMan Gene Expression Assays (Thermo Fisher Scientific, Inc., Waltham, MA, USA) were performed with the use of a ViiA™7 Real-Time PCR system (Applied Biosystems; Thermo Fisher Scientific, Inc.) under the following thermocycling conditions: 48 °C for 15 min, 95 °C for 10 min; and 40 cycles of 95 °C for 15 s and 60 °C for 1 min. The TaqMan Probes used for qRT-PCR are presented in Table 1. The data were normalized to the reference genes (*GAPDH* and *ACTB*, Table 1) and the relative expression level of each target gene compared to the control group was expressed as 2^−ΔΔCt^. All qRT-PCR experiments were run in triplicate, and the mean value was used for the determination of mRNA levels [14].

### 2.7. Statistical Analysis of the Obtained Research Results

Statistical analyses were performed using the statistical package PQStat, version 1.8.2.212. 

The results of the study were compared using analysis of variance (ANOVA) and Tukey’s post hoc test. A test probability of *p* < 0.05 was considered significant, and a test probability of *p* < 0.01 was considered highly significant.

## 3. Results

### 3.1. Effects of Nano-, Micro-, and Macrogenistein on the Development and Progression of DMBA-Induced Tumorigenesis in Rats

The study showed that the incidence of tumors was 100% in all study groups except for the microgenistein-supplemented animals (88%) (Table 2). The first palpable tumors appeared in the group of nanogenistein-supplemented animals as early as 14 weeks of age. For the unsupplemented animals, the first tumors in the group appeared at week 16 (2 weeks later for nanogenistein-supplemented animals), and for the micro- and macrogenistein-supplemented animals at week 17 (3 weeks later for nanogenistein-supplemented animals). The numbers of tumors (assessed at week 20) per individual varied according to the supplementation used, and were for micro: 0–3; nano: 2–5; macro: 1–6, and for the control group: 2–9. Interestingly, the weight of tumors was significantly higher for rats supplemented with microgenistein (mean ± SD: 1.99 ± 1.75; range: 0.11–6.11) and nanogenistein (mean ± SD: 1.59 ± 2.64; range: 0.06–9.50), relative to animals without supplementation (mean ± SD: 0.93 ± 1.34; range: 0.10–7.80).

**Figure 1:**
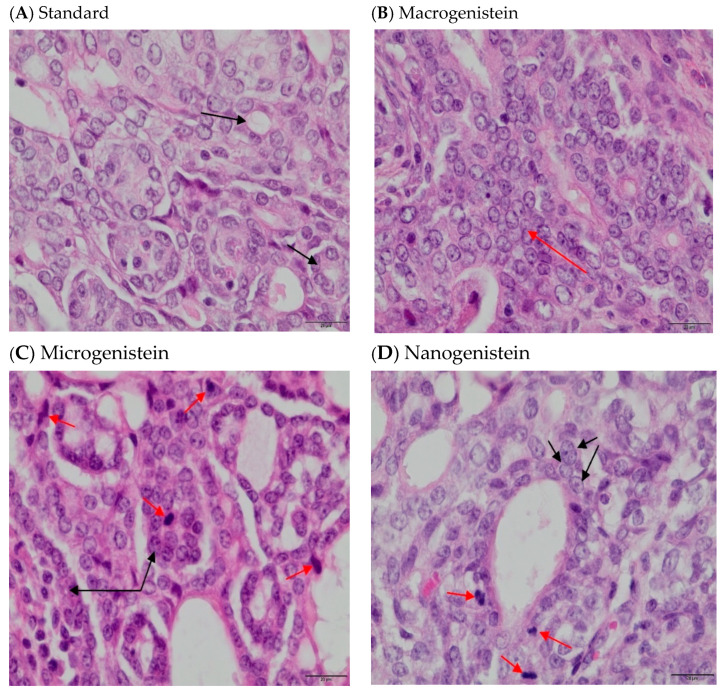
Hematoxylin-and-eosin-stained sections of breast tumors. (**A**) rats fed with a standard diet—no supplementation (black arrows show the tubular system of tumor cells); (**B**) rats fed with nanogenistein (red arrow indicates infiltrating tumor cell); (**C**) rats fed with a diet supplemented with microgenistein (red arrows indicate mitosis and black bands indicate tumor cells); (**D**) rats fed with a diet supplemented with macrogenistein (red arrows show mitosis and black arrows show cancer cells forming vesicles) [11].

Histopathological examination showed that all tumors examined had features of breast cancer—adenocarcinoma (Figure 1, Table 3). A grade II adenocarcinoma was found in animals without supplementation and in animals supplemented with macrogenistein. In tumor samples obtained from microgenistein-supplemented and nanogenistein-supplemented animals, the histopathological examination image indicated grade III malignancy. The groups of animals that were supplemented with genistein showed an increase in the intensity of tumor cell proliferation, as evidenced by the number of mitoses in the field of view of the microscope (at 40× objective magnification), with respect to animals without supplementation (Table 3). The mean numbers of mitoses in genistein-supplemented animals was statistically significantly higher compared to the control animals (mean ± SD: 1.79 ± 1.25), and were mean ± SD: 7.33 ± 1.57 (microgenistein), mean value ± SD: 5.82 ± 1.57 (nanogenistein), and 4.46 ± 2.38 (macrogenistein) [11].

### 3.2. The Effect of Nano-, Micro-, and Macrogenistein Supplementation on the Body Weight of the Test Animals and the Weight of Their Organs

**Figure 2:**
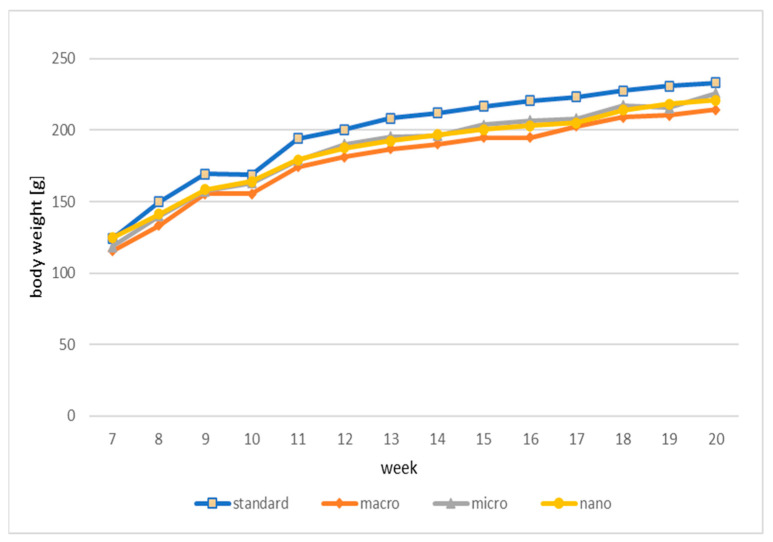
Kinetics of changes (weeks 7–20) in body weight (g) of experimental animals. Standard: animals receiving a standard diet only (no supplementation); macro: animals supplemented with macrogenistein; micro: animals supplemented with microgenistein; nano: animals supplemented with nanogenistein; g: grams; differences not statistically significant (α = 0.05).

The data on the kinetics of the changes (7–20 weeks of age of animals) in body weight (g) of animals treated with DMBA and supplemented with genistein in macro, micro, and nano forms and animals without supplementation are shown in Figure 2.

**Figure 3:**
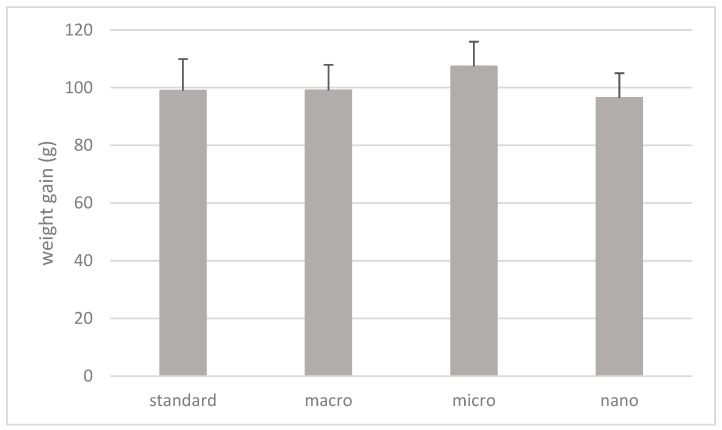
Weight gain of rats (g). Standard: animals without supplementation; macro: animals supplemented with macrogenistein; micro: animals supplemented with microgenistein; nano: animals supplemented with nanogenistein; g: grams; differences not statistically significant (α = 0.05).

The results of the statistical analysis of the body weight gain of rats (7–20 week old rats) depending on the supplementation used are shown in Figure 3.

Based on the results, there were no statistically significant differences in the weight gain of animals (g) over a period of 13 weeks (from 7 to 20 weeks of age) depending on the supplementation used (Figure 3).

**Figure 4:**
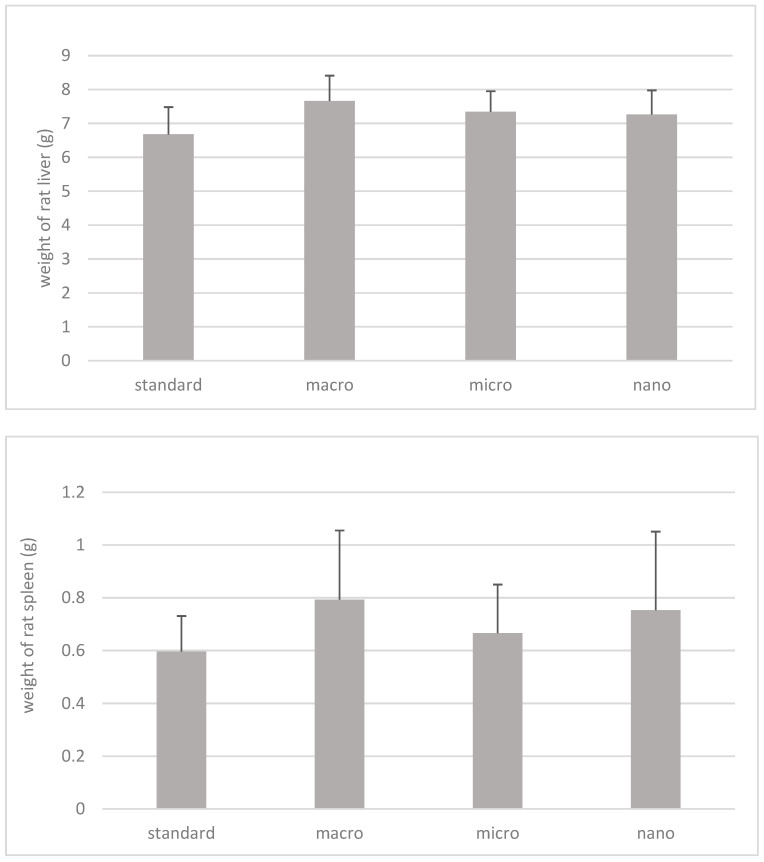
Weight of rats’ spleen and liver (g). Standard: rats without supplementation; macro: animals supplemented with macrogenistein; micro: animals supplemented with microgenistein; nano: animals supplemented with nanogenistein; g: grams; differences not statistically significant (α = 0.05).

The results of a statistical analysis of organ weights (g), liver and spleen of animals supplemented with nano-, micro-, and macrogenistein in relation to animals without supplementation, are shown in Figure 4. The material for the study was obtained at 20 weeks of age of the animals, during their decapitation.

Based on the study, it was shown that the weights of spleens and livers of rats supplemented with macro-, micro-, and nanogenistein were higher with respect to animals without supplementation (control group); however, due to the high values of the standard deviation, these values were not statistically significant (Figure 4).

### 3.3. Effects of Nano-, Micro-, and Macrogenistein on the Content of Selected Fatty Acid Metabolites in the Serum of Rats Treated with DMBA

The results of 5-, 12-, and 15-hydroxyyeicosatetraenoic acids and hydroxyoctadecadienoic acids in the blood serum of rats treated with DMBA and supplemented with nano-, micro-, and macrogenistein are shown in Table 4.

Based on the study, there was a statistically significant lower content of 12-HEPE (*p* = 0.001); HODE (*p* = 0.0001), and 12-HETE (*p* = 0.0001) in the serum of rats supplemented with genistein in the macro, micro, and nano forms, with respect to the content of the markers in the serum of rats without supplementation. There were no statistically significant differences in the content of 5-HETE and 15-HETE acids in the serum of rats depending on the supplementation used (Table 4).

### 3.4. Effects of Nano-, Micro-, and Macrogenistein on the Contents of Interleukin-1, Interleukin-6, and Metalloproteinase-9 in the Serum of Rats Treated with DMBA

The results of the contents of interleukin-1, interleukin-6, and metalloproteinase-9 in the serum of animals treated with a carcinogenic agent and supplemented with nano-, micro-, or macrogenistein are shown in Table 5.

Based on the results, there were no statistically significant differences in the levels of IL-1, IL-6, and MMP-9 in the serum of genistein-supplemented rats, relative to animals without supplementation.

### 3.5. Effects of Nano-, Micro-, and Macrogenistein on Gene Expression of Interleukin-1, Interleukin-6, and Metalloproteinase-9 in the Blood Serum of Rats Treated with DMBA

**Figure 5:**
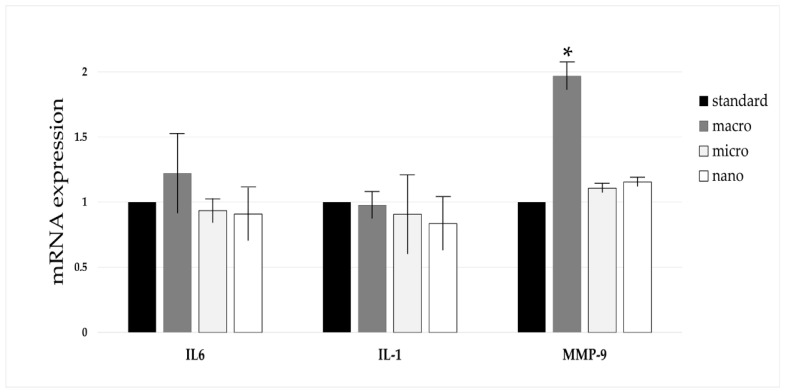
Results of gene expression of interleukin-1, interleukin-6 and metalloproteinase-9 in blood of animals treated with (DMBA) and supplemented with nano-, micro-, or macrogenistein; standard: animals without supplementation; macro: animals supplemented with macrogenistein; micro: animals supplemented with microgenistein; nano: animals supplemented with nanogenistein. The results were expressed as the relative quantification (the fold of standard group). * *p* < 0.05 compared to standard group.

The results of the gene expression of *IL-1, IL-6,* and *MMP-9* in the blood of animals treated with DMBA and supplemented with nano-, micro-, or macrogenistein are shown in Figure 5.

On the basis of the study, we found a significant increase in the expression of the *MMP-9* gene in the blood of rats supplemented with macrogenistein (compared to animals without supplementation or supplemented with micro- or nanogenistein). The expression of the IL-1 gene in the blood of nano- and microgenistein-supplemented rats was shown to be reduced, relative to animals without supplementation; however, this did not reach statistical significance.

## 4. Discussion

Based on the study, it was shown that supplementation of animals with genistein in macro, micro, and nano forms increased the intensity of the tumor process in rats. Histopathological examination showed that all tumors examined had features of breast cancer—adenocarcinoma. A grade II adenocarcinoma was found in animals without supplementation and in animals supplemented with macrogenistein. In tumor samples obtained from microgenistein-supplemented and nanogenistein-supplemented animals, the histopathological examination image indicated grade III malignancy. The groups of animals that were supplemented with genistein showed an increase in the intensity of tumor cell proliferation, as evidenced by the number of mitoses in the field of view of the microscope (at 40× objective magnification), with respect to animals without supplementation. Macro-, micro-, and nanogenistein caused an increase in the intensity of tumor cell proliferation. Higher proliferative potential is associated with a poor prognosis and indicates rapid proliferation of tumor cells [15]. Ju Y.H. et al. [7] showed that the carcinogenic potential of genistein depended on the dose of genistein. The use of higher concentrations of this compound was associated with breast tumor growth, increased cell proliferation, and pS2 expression. Reducing materials to the nanoscale can sometimes lead to the development of new structural, phytochemical, electronic, and magnetic properties that are not present in larger particles containing the same material. Nanoparticles acquire new physical properties by increasing the surface-to-volume ratio, and having different reactive sites, charge, shape, mobility, and thermal properties. The form of the particles can unambiguously change their properties, and thus, the effect of nanoparticles on biological activity [16]. For this reason, these compounds are used in the diagnosis and treatment of various diseases, allowing earlier detection of pathological changes and more effective treatment of patients [17]. So far, clinical studies and observations indicate that the effect of genistein is inconclusive—some show it to have a protective effect in cancer, while others show it to be carcinogenic. However, given the structure of genistein and its proven potential to enhance the proliferation of some cancer cells, particularly in hormone-dependent cancers, the use of particle size reduction in this study exacerbated cancer. The best known property of genistein is its oestrogenic activity [7,8,18,19]. Genistein is a relatively strong agonist of the oestrogen receptor beta isoform (ERβ). Owing to its structural similarity to oestrogen, genistein shows similar activity to oestrogen [20]. However, the role of genistein in breast cancer can be determined by multiple factors, including age-dependent biological effect, the ratio of alpha and beta oestrogen receptors, gene mutations, individual differences in metabolism, the possibility of action through various metabolic pathways, and inflammation [8,21,22]. Considering that the role of genistein in breast cancer is ambiguous, learning about the mechanisms of action of genistein based on the analysis of selected biomarkers seems to be of great importance in assessing the safety of its use. 

Inflammation is one of the factors in the initiation and progression of the cancer process [23,24]. Fatty acids are subject to metabolic processes that produce substances with the character of modulators of immune responses and inflammation, these include, among others, derivatives of hydroxyeicosatetraenoic, hydroxyoctadecadienoic, and hydroxyeicosapentaenoic acids. Arachidonic acid, metabolized by the enzymes 5-, 12-, and 15-lipoxygenase (5-LOX, 12-LOX, 15-LOX), is converted into hydroxyeicosatetraenoic acids: 5-HETE, 12-HETE, and 15-HETE, respectively. HODE are formed by the metabolism of linoleic acid. The oxidation of linoleic acid via 12-LOX and 15-LOX produces 9-hydroxyoctadecadienoic acid (9-HODE) and 13-hydroxyoctadecadienoic acid (13-HODE). With the participation of 12-lipoxygenase, 12-hydroxyeicosapentaenoic acid (12-HEPE) is formed from eicosapentaenoic acid [25,26]. The above-mentioned fatty acid metabolites, even in very low concentrations, are characterized by high biological activity [24,25,26,27]. They can play an important role in the development and course of a number of diseases, including atherosclerosis, cardiovascular diseases, allergies, autoimmune diseases, or cancer. The direction of this action depends on the type of compounds formed and the cascade of reactions in which they are involved [25,26]. Based on our study, we found a statistically significantly lower content of 12-HEPE (*p* = 0.001), HODE (*p* = 0.0001), and 12-HETE (*p* = 0.0001) in the blood serum of genistein-supplemented rats, with respect to the content of the aforementioned markers in the serum of rats without supplementation. HEPE acids are considered a possible modulator of the tumor process. Treatment of cancer cells with HEPE results in inhibition of their growth. 12-HEPE has been found to inhibit the uptake of 3H-thymidine (a marker of cell proliferation and growth) by cancer cells. It has been shown to modulate cell proliferation and apoptosis [28]. 13-HODE acid can affect the development and progression of cancer by regulating cancer cells’ motility and their adhesion and migration through capillaries in the endothelium. If cancer cells are unable to adhere to the endothelium and migrate across the barrier, it hinders the stabilization of metastatic foci. It has been shown that tumor cells producing small amounts of 13-HODE are characterized by easier adhesion to the endothelium. In a study by Tavakoli-Yaraki et al. [29], 13-HODE was found to inhibit the growth of both MCF-7- and MDA-MB-231-type cancer cells. It causes cell cycle arrest and induces apoptosis and decreases the level of PPAR-δ receptor expression. Both 15-LOX expression and HODE levels have been shown to be downregulated in cancer cells [30]. Breast cancer patients have significantly reduced levels of 15-LOX in tumor-transformed breast tissues, relative to levels in breast tissues obtained from healthy women [31,32]. It is difficult to explain the decrease in serum levels of 12-HETE in the rats studied, for it is a compound with inhibitory effects on apoptosis, promoting tumor angiogenesis and adhesion of tumor cells to endothelial cells [33]. Perhaps this result is due to the inhibitory effect of genistein on both 15- and 12-lipoxygenase activity [34].

Another objective of the current study was to evaluate the effects of nano-, micro-, and macrogenistein on the content of interleukin-6 (IL-6), interleukin-1 (IL-1), and metalloproteinase-9 (MMP-9) in the serum of DMBA-treated rats. The effect of genistein supplementation on the expression of genes encoding selected compounds in the blood of rats was also evaluated. Interleukins are proteins belonging to the cytokine group [35,36]. Cytokines are responsible for communication between cells. They condition the interaction of cells. At physiological concentrations, interleukins play an important role in cell proliferation, maturation, migration, and adhesion. They have been shown to influence metabolic processes and the neuroendocrine system, thereby taking part in maintaining homeostasis in the body [37]. However, interleukins may also play an important role in tumorigenesis, including initiation of carcinogenesis, angiogenesis, and metastasis [38,39,40]. Elevated levels of IL-1 interleukins have been shown to occur in the body fluids of cancer patients, relative to healthy individuals [41]. Local or systemic overexpression of the gene encoding IL-6 has been found in cancer patients [40,41]. A study by Kozlowski et al. [40] showed higher serum levels of IL-6 in patients with stage III disease, relative to patients with stage II disease. Elevated serum levels of IL-1 and/or IL-6 in patients are usually associated with poor prognosis and poor survival of breast cancer patients [41,42]. It has been shown that the levels of interleukin family 1 and IL-6 are significantly higher in tumor-lesioned tissue compared to healthy tissue [38]. However, based on this study, there were no statistically significant differences in the content or expression of IL-1 and IL-6 genes in the blood of DMBA-treated rats, depending on the supplementation used. Tang et al. [43] showed that genistein inhibits gene expression and prevents IL-1 formation in UVB-treated keratinocytes. Lee et al. [44] showed that genistein supplementation of diethylnitrosamine (DEN)-treated mice results in decreased IL-6 and TNF gene expression (relative to control animals). The anti-inflammatory effects of genistein have been demonstrated in a number of studies [45]. Interestingly, it was found that animals supplemented with nano-, micro-, and macrogenistein had higher levels of metalloproteinase-9, MMP-9, compared to animals without supplementation. There was a significant increase in MMP-9 gene expression in the blood of macrogenistein-supplemented animals. Extracellular matrix metalloproteinases are a group of metal-dependent proteolytic enzymes belonging to the group of endopeptidases, involved in the degradation of basement membrane proteins and extracellular matrix, which enables tissue remodeling and cell movement, both in the course of physiological processes, inflammation, and cancer. MMP-9 regulates the activity of growth factors, cytokines and especially chemokines [46]. Metalloproteinase-9 plays an important role in tumorigenesis [47]. It is characterized by its ability to degrade type IV collagen, and thus, participates in the mechanism of damage to the vascular basement membrane. This feature determines the process of angiogenesis, local tumor growth, and the formation of metastases. Once the tumor crosses the basement membrane of blood vessels, it gains the ability to form metastases in areas distant from the primary focus. In breast cancer, metalloproteinases have been found to increase in concentration and activity with tumor progression. Their increased expression in the tumor infiltrate is considered a new prognostic factor, but also a factor monitoring the effectiveness of therapy in the course of cancer [47]. 

## 5. Conclusions

In conclusion, it was shown that supplementation of animals with nano-, micro-, and macrogenistein had an effect on both the development of the tumor process, as well as on the concentrations of selected eicosanoids (HEPE, HODE, HETE) in the serum of rats treated with 7,12-dimethylbenzanthracene. It was found that animals supplemented with nano-, micro-, and macrogenistein had higher levels of metalloproteinase-9, compared to animals without supplementation. There was a significant increase in *MMP-9* gene expression in the blood of macrogenistein-supplemented animals, relative to the other groups of rats. Given the role that inflammation may play in the development and progression of the cancer process, further research in this direction is needed, taking into account both a larger number of rats and a broader panel of biomarkers to be studied.

## Figures and Tables

**Table 1:** TaqMan probes used for qRT-PCR in determination of the gene expression of selected angiogenic factors.

Gene Name	Gene Symbol	Assay ID
Matrix metallopeptidase-9	*MMP-9*	Rn00579162_m1
Interleukin-1 beta	*IL-1b*	Rn00580432_m1
Interleukin-6	*IL-6*	Rn01410330_m1
Glyceraldehyde-3-phosphate dehydrogenase	*GAPDH*	Rn01775763_g1
Actin beta	*ACTB*	Rn00667869_m1

**Table 2:** Cancer induction in 7,12-dimethylbenz[a]anthracene-treated rats in relation to supplementation [11].

Supplementation	Rat’s Number	Tumor Weight (g) (Mean ± SD) (Week 20)	Number of Tumors per Rat (Week 20)	Incidence of Tumors per Rat in Time (Weeks of Rats Life)
Standard	1	0.90 ± 0.78 (0.1–2.7)	9	17
	2	1.84 ± 3.35 (0.1–7.8)	5	16
	3	0.43 ± 0.30 (0.1–0.8)	4	19
	4	0.45 ± 0.40 (0.1–1.1)	6	18
	5	1.02 ± 1.03 (0.1–2.6)	6	16
	6	0.60 ± 0.00 (0.6)	2	19
	7	1.36 ± 0.92 (0.3–1.96)	3	17
	8	0.61 ± 0.20 (0.47–0.75)	2	17
	mean ± SD	0.93 ± 1.34 (0.10–7.80) ^a^		
Macrogenistein	1	2.35 ± 1.96 (0.9–5.14)	4	17
	2	0.52 ± 0.47 (0.14–1.38)	6	17
	3	0.70 ± 0.52 (0.33–1.29)	3	19
	4	1.83 ± 2.48 (0.13–6.39)	6	18
	5	1.74 ± 1.79 (0.24–4.07)	4	17
	6	0.34	1	19
	7	1.58 ± 1.14 (0.29–2.42)	3	17
	8	1.13 ± 1.28 (0.25–3.03)	4	17
	mean ± SD	1.27 ± 1.52 (0.14–6.39)		
Microgenistein	1	0.89 ± 0.40 (0.6–1.17)	2	20
	2	-	-	-
	3	6.11	1	18
	4	1.38 ± 0.69 (0.89–1.86)	2	20
	5	3.31	1	17
	6	2.77 ± 1.81 (0.69–3.89)	3	18
	7	1.45 ± 1.40 (0.19–2.96)	3	18
	8	0.66 ± 0.78 (0.11–1.21)	2	19
	mean ± SD	1.99 ± 1.75 (0.11–6.11) ^a^		
Nanogenistein	1	1.01 ± 1.02 (0.1–2.43)	4	19
	2	4.58 ± 2.12 (3.08–6.08)	2	17
	3	0.28 ± 0.20 (0.09–0.48)	3	19
	4	0.20 ± 0.07 (0.1–0.26)	4	20
	5	1.92 ± 3.74 (0.11–8.61)	5	14
	6	1.57 ± 1.94 (0.06–4.41)	4	18
	7	5.03 ± 6.32 (0.56–9.50)	2	18
	8	0.30 ± 0.16 (0.18–0.41)	2	20
	mean ± SD	1.59 ± 2.64 (0.06–9.50)		

SD—standard deviation; ^a^—statistically significant differences between groups (*p* < 0.01).

**Table 3:** Histopathological examination of rats’ tumors.

Supplementation	Tumor Grade	The Mean Number of Mitoses in the Field of View Area *
Standard	Adenocarcinoma grade 2	1.79 ± 1.25 ^a,b,c^
Macrogenistein	Adenocarcinoma grade 2	4.46 ± 2.38 ^a,d^
Microgenistein	Adenocarcinoma grade 3	7.33 ± 1.57 ^b,d^
Nanogenistein	Adenocarcinoma grade 3	5.82 ± 1.57 ^c^

Data are expressed as mean ± SD (standard deviation). Values sharing letters (^a^: standard, ^b^: macrogenistein, ^c^: microgenistein, ^d^: nanogenistein) indicate statistically significant differences between groups (*p* < 0.01). * Mitoses were counted in slides from randomly selected tumors in 15 fields of view with a 40× objective magnification.

**Table 4:** The levels of 5-, 12-, and 15-hydroxyeicosatetraenoic acids, 12-hydroxyeicosapentaenoic acid, and the sum of hydroxyoctadecadienoic acids (ng/mL) in the serum of rats treated with a carcinogen and supplemented with macro-, micro-, or nanogenistein.

Eicosanoids	Group				*p*-Value
	Standard	Macro	Micro	Nano	
12-HEPE	458.19 ± 208.09 ^b^	69.45 ± 36.42 ^a^	61.07 ± 12.68 ^a^	60.21 ± 14.18 ^a^	0.0013
HODE	226.82 ± 33.67 ^b^	110.60 ± 48.13 ^a^	130.52 ± 41.90 ^a^	116.13 ± 32.58 ^a^	0.0001
15-HETE	43.10 ± 7.58 ^b^	29.06 ± 6.49 ^a^	36.82 ± 14.09 ^ab^	36.18 ± 8.68 ^ab^	n.s.
12-HETE	6446 ± 1522 ^b^	2414 ± 917 ^a^	1918 ± 219 ^a^	1897 ± 319 ^a^	0.0001
5-HETE	10.94 ± 2.81	6.84 ± 3.64	9.29 ± 6.25	15.73 ± 14.17	n.s.

Data are presented as mean values ± standard deviation (SD); standard: animals without supplementation; macro: animals supplemented with macrogenistein; micro: animals supplemented with microgenistein; nano: animals supplemented with nanogenistein; *p*-value: test probability (statistical significance); ^a,b^—homogeneous groups in rows (α = 0.05); n.s.—differences not statistically significant (α = 0.05); 12-HEPE—12-hydroxyyeicosapentaenoic acid; HODE—sum of hydroxyoctadecadienoic acids; 15-HETE—15-hydroxyyeicosatetraenoic acid; 12-HETE—12-hydroxyyeicosatetraenoic acid; 5-HETE—5-hydroxyyeicosatetraenoic acid; ng: nanogram; ml: milliliter.

**Table 5:** Levels of interleukin-6 (IL-6) (pg/mL), interleukin-1 (IL-1) (pg/mL), and metalloproteinase-9 (MMP-9) (ng/mL) in serum of rats treated with DMBA and supplemented with macro-, micro-, or nanogenistein.

Eicosanoids	Group				*p*-Value
	Standard	Macro	Micro	Nano	
IL-6	88.47 ± 40.39	109.97 ± 57.64	99.36 ± 43.12	92.01 ± 16.50	n.s.
IL-1	103.64 ± 69.86	86.05 ± 35.07	104.25 ± 32.82	132.43 ± 65.78	n.s.
MMP-9	0.64 ± 0.35	1.67 ± 1.10	1.80 ± 1.05	2.03 ± 2.68	n.s.

Data are presented as mean values ± standard deviation (SD); standard: animals without supplementation; macro: animals supplemented with macrogenistein; micro: animals supplemented with microgenistein; nano: animals supplemented with nanogenistein; *p*-value: test probability (statistical significance); n.s.—differences not statistically significant (α = 0.05); Il-6—interleukin-6; IL-1—interleukin-1; MMP-9—metalloproteinase-9.

## Data Availability

The datasets generated for this study are available on request to the corresponding author.
